# Malaria Clusters among Illegal Chinese Immigrants to Europe through Africa

**DOI:** 10.3201/eid0909.030353

**Published:** 2003-09

**Authors:** Zeno Bisoffi, Alberto Matteelli, Donatella Aquilini, Giovanni Guaraldi, Giacomo Magnani, Giovanna Orlando, Giovanni Gaiera, Tomas Jelinek, Ron H. Behrens

**Affiliations:** * Ospedale S. Cuore, Negrar, Verona, Italy; † Università di Brescia, Brescia, Italy; ‡ Ospedale di Prato, Prato, Italy; § Università di Modena, Modena, Italy; ¶ Ospedale di Reggio Emilia, Reggio Emilia, Italy; # Ospedale Sacco, Milano, Italy; ** Ospedale S. Raffaele, Milano, Italy; †† Institute of Tropical Medicine, Berlin, Germany; ‡‡ London School of Hygiene and Tropical Medicine, London, United Kingdom

**Keywords:** Malaria, Illegal migrants, China, Europe, Italy, Cote d’Ivoire, SARS virus, travel

## Abstract

Between November 2002 and March 2003, 17 cases of malaria (1 fatal) were observed in illegal Chinese immigrants who traveled to Italy through Africa. A further cluster of 12 was reported in August, 2002. Several immigrants traveled by air, making the risk of introducing sudden acute respiratory syndrome a possibility should such illegal immigrations continue.

From November 2002 to March 2003, 17 cases of malaria were noted among illegal Chinese immigrants in seven hospitals across central and northern Italy (15 cases of *Plasmodium falciparum*, 1 case of *P. malariae*, and 1 mixed infection of *P. falciparum* and *P. malariae*). One patient died. Until recently, imported malaria in this group of illegal immigrants from China was not detected by malaria surveillance institutions within Europe (*1*). Although malaria is still endemic in parts of China, transmission in these regions is low-level (*2*); the predominant species is *P. vivax*. *P. falciparum* transmission is confined to provinces bordering Laos and Viet Nam. None of the patients reported coming from those areas. Investigating the cluster proved difficult because of language problems and reticence to provide detailed information of travel, since the patients were illegal immigrants (Table). The fatal case occurred in a general hospital in northern Italy. The 20-year-old woman (case 7) was admitted with a high fever, severe hemolytic anemia (hemoglobin 4.4 g/dL), and metabolic acidosis. After 48 hours, because of hypotension, seizures, and subsequent coma, she was transferred to the intensive-care unit of a referral hospital for infectious diseases. The blood film showed a 70% parasitemia with *P. falciparum.* The patient died 96 hours after admission, despite aggressive drug therapy and plasmapheresis.

**Table:** Characteristics of 17 cases of malaria in illegal Chinese immigrants, Italy

Case	Sex, age^a^	Date first seen by physician	Country of transit	Time spent in country of transit	Mode of travel	Mode of travel to Europe	*Plasmodium* species	Clinical outcome
1	M, 21	11/05/02	Côte d’Ivoire	8 mo	Air	Air	*P. falciparum*	Recovered
2	M, 24	11/11/02	“Africa”	3 mo	Unknown	Air	*P. falciparum*	Recovered
3	F, 20	11/12/02	Côte d’Ivoire	22 d	Road/sea	Air	*P. falciparum*	Recovered
4	M, 22	11/15/02	Côte d’Ivoire	1 mo	Air	Air	*P. falciparum*	Recovered
5	M, 24	11/16/02	Côte d’Ivoire	14 d	Road/sea	Air	*P. falciparum*	Recovered
6	M, 28	01/09/03	Côte d’Ivoire	2 mo	Unknown	Air	*P. falciparum*	Recovered
7	F, 20	01/13/03	Côte d’Ivoire	Few days	Unknown	Air	*P. falciparum*	Died
8	M, 21	02/01/03	Côte d’Ivoire	Unknown	Unknown	Air	*P. falciparum*	Recovered
9	F, 32	02/02/03	Congo	Unknown	Unknown	Air	*P. falciparum*	Recovered
10	M, 22	02/03/03	Côte d’Ivoire	6 mo	Air	Air	*P. falciparum*	Recovered
11	M, 19	02/08/03	Côte d’Ivoire	Unknown	Unknown	Air	*P. falciparum*	Recovered
12	M, 34	02/13/03	Congo	2 mo	Road/sea	Air	*P. falciparum* and *P. malariae*	Recovered
13	F, 24	02/13/03	Côte d’Ivoire	50 d	Air	Air	*P. falciparum*	Recovered
14	M, 40	02/22/03	Côte d’Ivoire	Unknown	Road/sea	Air	*P. falciparum*	Recovered
15	M, 22	02/24/03	Côte d’Ivoire	2 mo	Road/sea	Air	*P. falciparum*	Recovered
16	M, 28	03/01/03	“Africa”	Unknown	Unknown	Air	*P. falciparum*	Recovered
17	M, 23	03/15/03	Côte d’Ivoire	50 d	Road/sea	Air	*P. malariae*	Recovered

^a^M, male; F, female.

## Discussion

Before 2000, no cases of *P. falciparum* had occurred in Chinese immigrants living in northern and central Italy, despite a large immigrant population. An initial cluster of 22 cases was described during summer 2000 in the Lombardy Region (*3*). A cluster of six cases was detected in Tuscany during the same period (*4*). In both outbreaks, the researchers described high rates of severe disease. All patients were exposed to malaria during a prolonged journey to Europe (3–9 months) through a number of Asian and African countries.

From 2000 to 2002, a total of 10 sporadic cases were reported to the Italian Ministry of Health in 2001 (L. Vellucci, Directorate for Prevention, Ministry of Health, Italy, pers. comm.). The 2003 cluster prompted us to examine hospital records from August 2002, where we identified an additional, previously undetected, cluster of 12 malaria cases in four of our study hospitals (data not included in the table). The Ministry of Health had 26 confirmed *P.* *falciparum* cases during 2002 (L. Vellucci, pers. comm.), suggesting an ongoing (and possibly increasing) influx of Chinese laborers. Some differences exist between the later cluster and the 2000 cluster. In the 2003 cluster, the proportion of severe cases was lower than in the previous reports, with a patient with a fatal case first admitted to a general hospital where diagnosis of malaria was not considered; in the others, awareness of the possibility of malaria had been raised by the earlier cluster (*3*,*4*) and led to prompt diagnosis and treatment, with favorable outcome. A single African country, Côte d’Ivoire, was the transit country for most of the patients. In previous cases, a number of other African countries were used for transit. Visa processing for entry to Europe was arranged by the courier organization in Côte d’Ivoire. The clustering of cases suggests that the illegal immigrants arrive in Europe in groups. Although Italy was the final destination, at least some immigrants entered through France, which also has had reports of *P. falciparum* cases in Chinese immigrants (F. Legros, Centre National de Référence de l’Epidémiologie du Paludisme, France, pers. comm.). As malaria is probably underreported in Europe, additional cases may well have occurred.

Use of clandestine travel by air to emigrate from China, where sudden acute respiratory syndrome (SARS) is present, poses a threat for the African countries, where the introduction of SARS virus could have devastating consequences on their health systems with a potential overlap with the HIV epidemic. Other diseases could be spread or acquired by the immigrants in the countries of transit. While curtailing the huge, illegal immigrant system to Europe is difficult, we cannot overemphasize the need for a sound surveillance on imported infectious diseases in this continent.

Both clusters of malaria were detected early through Salute Internazionale Regione Lombardia (SIRL), a network on imported diseases of the Lombardy Region, in conjunction with the European Network on Imported Infectious Disease Surveillance (TropNetEurop). Any physician in Europe who sees a Chinese patient with a history of recent travel and a high fever should exclude malaria, besides considering the possible diagnosis of SARS. Respiratory symptoms are also frequent in uncomplicated malaria (*5*,*6*), and acute respiratory distress syndrome has long been recognized as one of the main features of severe malaria (*7*,*8*).
